# The influence of both individual and area based socioeconomic status on temporal trends in Caesarean sections in Scotland 1980-2000

**DOI:** 10.1186/1471-2458-11-330

**Published:** 2011-05-18

**Authors:** Lesley Fairley, Ruth Dundas, Alastair H Leyland

**Affiliations:** 1 Bradford Institute for Health Research, Bradford Royal Infirmary, Duckworth Lane, Bradford, BD9 6RJ, UK; 2 MRC/CSO Social and Public Health Sciences Unit, 4 Lilybank Gardens, Glasgow, G12 8RZ, UK

**Keywords:** Caesarean section, social class, area deprivation

## Abstract

**Background:**

Caesarean section rates have risen over the last 20 years. Elective Caesarean section rates have been shown to be linked to area deprivation in England, women in the most deprived areas were less likely to have an elective section than those in the most affluent areas. We examine whether individual social class, area deprivation or both are related to Caesarean sections in Scotland and investigate changes over time.

**Methods:**

Routine maternity discharge data from live singleton births in Scottish hospitals from three time periods were used; 1980-81 (n = 133,555), 1990-91 (n = 128,933) and 1999-2000 (n = 102,285). Multilevel logistic regression, with 3 levels (births, postcode sector and Health Board) was used to analyse emergency and elective Caesareans separately; analysis was further stratified by previous Caesarean section. The relative index of inequality (RII) was used to assess socioeconomic inequalities.

**Results:**

Between 1980-81 and 1999-2000 the emergency section rate increased from 6.3% to 11.9% and the elective rate from 3.6% to 5.5%. In 1980-81 and 1990-91 emergency Caesareans were more likely among women at the bottom of the social class hierarchy compared to those at the top (RII = 1.14, 95%CI 1.00-1.25 and RII = 1.13, 1.03-1.23 respectively) and also among women in the most deprived areas compared to those in the most affluent (RII = 1.18, 1.05-1.32 and RII = 1.13, 1.02-1.26 respectively). In 1999-2000 the odds of an elective section were lower for women at the bottom of the social class hierarchy than those at the top (RII = 0.87, 0.76-1.00) and also lower in women in the most deprived areas compared to those in the most affluent (RII = 0.85, 0.73-0.99).

**Conclusions:**

Both individual social class and area deprivation are independently associated with Caesarean sections in Scotland. The tendency for disadvantaged women to be more likely to receive emergency sections disappeared at the same time as the likelihood of advantaged groups receiving elective sections increased.

## Background

Caesarean section rates have risen throughout the world over the last 20 years. In Spain the rate was 17.6% in 2000, [1] and the rate was 15.4% in 2004 in Norway [2]. The Caesarean rate reached a high of 32% in the US in 2008 [3]. In Scotland it has increased from 11.6% in 1980 to 25.9% in 2008 [4] and in England it has risen from 9% in 1980 to 23% in 2003-04 [5]. These rates are outwith the 1985 WHO recommended rates of 5%-15% [6]. Increasing rates may be due to a variety of factors, with the tendency for women to delay childbirth potentially responsible for more than a third of this increase [7].

Rising rates of Caesarean section are concerning as a Caesarean section is major abdominal surgery and carries risks to the mother of thrombosis, excess bleeding and damage to the bladder [8]. There are also risks for babies born by Caesarean section. The most common problem affecting babies born by Caesarean section is breathing difficulties in the post-natal period [9]. Mothers who have delivered by Caesarean section have been shown to have increased hospital stays and morbidity, which in turn leads to an increase in costs to the provider [10,11].

The social patterning of Caesarean sections is unclear; a recent study from Norway found that women with the lowest level of education had the highest risk of Caesarean section [2]. In Spain women from a non-manual background were more likely to have had a Caesarean birth [12]. In the UK, studies suggest that women in the most deprived areas of England were less likely to have an elective section than those in the most affluent areas [13,14]. However, in the early 1990s in Scotland there was no difference in section rates between women from affluent and deprived areas [15]. All of these UK studies used area deprivation as a marker of socioeconomic status; this measure describes the area not the individual [16].

In Scotland, we have routinely collected maternity discharge data which is linked to the birth registers to enable us to examine the effect of individual social class and area deprivation on both emergency and elective Caesarean sections. We also examine how these relationships have changed over time.

## Methods

Routine data on all hospital births in Scotland during the periods 1980-81, 1990-91 and 1999-2000 were provided by Information Services, NHS National Services Scotland (ISD). Information about the pregnancy and delivery was obtained from the Scottish Morbidity Record 02 (SMR02). The SMR02 has achieved national coverage of 98% of all births [4]. These records were linked to the Registrar General's birth registrations to obtain the occupational social class of the father of the child or the mother of the child if the father's occupation was not present on the form. We used Carstairs score as the measure of area deprivation. Carstairs scores are derived by combining variables measured at the Census and are available for the years 1981, [17] 1991 [18] and 2001 [19]. Carstairs scores of area deprivation were linked to birth records through postcode of the mother's address at birth; births from 1980-81 using the 1981 Carstairs score, births from 1990-91 the 1991 Carstairs scores and births from 1999-2000 the 2001 Carstairs scores. These scores were converted into quintiles based on the total population of Scotland at each time period, quintile 5 containing residents of the most deprived areas and quintile 1 residents of the most affluent areas. Analysis was restricted to live singleton births only. There were 364,733 such births in total; 133,555 in 1980-81, 128,933 in 1990-91 and 102,285 in 1999-2000. Births that were breech presentation were excluded from analysis (4557 (3.4%) in 1980-81, 4340 (3.4%) in 1990-91 and 3679 (3.6%) in 1999-2000) as Caesarean section is the preferred mode of delivery for breech presentations [20].

The postcode of the address at birth was missing, wrongly recorded or outside Scotland on 10,536 (8.2%) records in 1980-81, 7227 (5.8%) in 1990-91 and 7915 (8.0%) in 1999-2000 and therefore we could not assign these records a Carstairs score, and excluded them from the analysis. The rates of Caesarean section in those excluded due to a missing postcode was similar to the rates in those included in the analysis.

Caesarean sections were coded as either emergency or elective. Other maternal factors that were adjusted for were maternal age (<20, 20-24, 25-29, 30-34, 35-39 and 40+), maternal height (<155 cm, 155-164 cm and >164 cm), parity (0, 1, 2 and 3+), marital status (married (including cohabiting), single and other (including divorced, widowed and not known)) and gestational age (<37 weeks, 37-40 weeks and >40 weeks). In 1980-81, 7664 records were excluded due to missing information on the other maternal factors, 12119 in 1990-91 and 20440 in 1999-2000. The majority of these records did not have maternal height recorded (6131 (5.7%) in 1980-81, 11,343 (9.4%) in 1990-91 and 20,427 (20.0%) in 1999-2000). The Caesarean rates of those included and excluded from analysis were similar.

In the analysis, 110,798 (83.0%) births from 1980-81, 105,247 (81.6%) births from 1990-91 and 70,667 (69.0%) births from 1999-2000 were used.

Scotland is split into approximately 1000 postcode sectors or part sectors and at the time these were nested within 15 Health Boards. Multilevel logistic regression was used to account for the correlations of outcomes within postcode sectors and Health Boards. There were three levels in the multilevel models - level 1 individual (births) nested within level 2 postcode sectors nested within level 3 Health Boards. Emergency and elective Caesareans were analysed separately and data from each time period were analysed separately.

To compare the changes in social class inequalities in Caesareans over time the relative index of inequality (RII) was used [21-23]. This takes into account the fact that the proportion of the population in each social class at each time point differs. Social class was defined hierarchically (I, II, IIINM, IIIM, IV, V and Undetermined (this group includes people with inadequate job description, never worked, housewives and students)). The undetermined social class contained a heterogeneous mix of people, but it was evident that they experienced the worst perinatal outcomes [24] so this group was placed at the bottom of the social class hierarchy when creating the RII. Each group was assigned a value between 0 and 1 depending on the proportion of the population with higher socioeconomic position than the midpoint of each group within the hierarchy. Socioeconomic position was then related to Caesarean sections through multilevel logistic regression. The resulting odds ratio compares the bottom of the social hierarchy to the top; the larger the RII the larger the inequality. A similar index was created for the Carstairs quintiles to assess area deprivation inequalities.

All multilevel models were adjusted for the important confounders maternal age, maternal height, parity, marital status and gestational age. Multilevel models were fitted for each outcome at each time period including social class alone, area deprivation alone and both social class and area deprivation. In addition to social class and deprivation, an interaction between social class and quintiles of deprivation was tested for in the full model. Previous Caesarean section is a risk factor for subsequent Caesarean section and the characteristics and management of these mothers differ from those with no previous Caesarean section [25,26]. Analysis was further stratified by whether or not the mother had had a previous Caesarean section.

## Results

The emergency Caesarean section rate in Scotland rose from 6.3% in 1980-81 to 8.5% in 1990-91 to 11.9% in 1999-2000. The rates of elective Caesareans rose from 3.6% in 1980-81 to 5.5% in 1999-2000 (Table 1).

**Table 1:** Caesarean section rates by year, Scotland 1980-2000

	1980-1981		1990-1991		1999-2000	
Number of births	110798		105247		70667	
Number of Caesareans (%)	10973	(9.9)	12995	(12.3)	12279	(17.4)
Number of emergency Caesareans (%)	6972	(6.3)	8982	(8.5)	8388	(11.9)
Number of elective Caesareans (%)	3998	(3.6)	4013	(3.8)	3891	(5.5)

For all models there was no significant interaction between deprivation quintile and social class.

Table 2 shows the effect of individual social class and area deprivation (expressed as RIIs) on the odds of having an emergency Caesarean in each time period for all women and stratified by prior Caesarean section. In 1980-81 and 1990-91 both individual social class and area deprivation were associated with emergency sections. The odds of having an emergency section were greater for women at the bottom of the social class hierarchy than those at the top and also greater among women in the most deprived areas compared to those in the most affluent. In the fully adjusted model the RIIs for social class were of similar magnitude in 1980-81 and 1990-91. Women at the bottom of the social class hierarchy were 14% more likely to have an emergency Caesarean than women at the top in 1980-81 (RII = 1.14, 95% CI 1.04-1.25) and 13% more likely in 1990-91 (RII = 1.13, 1.03-1.23). There was no significant association between emergency sections and either social class or deprivation in 1999-2000. Among women with no previous Caesareans the social class and area deprivation trends over time were similar to that for all births. However, among women who had previously had a Caesarean there were no significant associations with socioeconomic status at any of the time points.

**Table 2:** Relative index of inequality (95% CI) for emergency Caesarean sections for social class (SC) and area deprivation (AD), Scotland 1980-2000

	1980-81			1990-91			1999-2000		
	**Model SC**	**Model AD**	**Model SC+AD**	**Model SC**	**Model AD**	**Model SC+AD**	**Model SC**	**Model AD**	**Model SC+AD**
	**RII (95%CI)**	**RII (95%CI)**	**RII (95%CI)**	**RII (95%CI)**	**RII (95%CI)**	**RII (95%CI)**	**RII (95%CI)**	**RII (95%CI)**	**RII (95%CI)**

**All Births**									
Social Class	1.16 (1.06-1.28)		1.14 (1.04-1.25)	1.15 (1.05-1.25)		1.13 (1.04-1.23)	1.03 (0.94-1.13)		1.02 (0.93-1.12)
Area Deprivation		1.21 (1.08-1.35)	1.18 (1.05-1.32)		1.16 (1.05-1.28)	1.13 (1.02-1.26)		1.03 (0.93-1.13)	1.02 (0.93-1.13)
**No previous Caesarean section**									
Social Class	1.18 (1.06-1.31)		1.15 (1.03-1.28)	1.18 (1.07-1.29)		1.15 (1.05-1.26)	1.04 (0.94-1.15)		1.04 (0.93-1.15)
Area Deprivation		1.26 (1.11-1.42)	1.23 (1.08-1.39)		1.20 (1.08-1.34)	1.17 (1.05-1.31)		1.03 (0.92-1.14)	1.02 (0.91-1.14)
**Previous Caesarean section**									
Social Class	1.25 (0.97-1.61)		1.24 (0.96-1.61)	1.03 (0.82-1.30)		1.03 (0.81-1.30)	0.90 (0.70-1.17)		0.90 (0.70-1.17)
Area Deprivation		1.07 (0.80-1.43)	1.02 (0.76-1.37)		1.01 (0.78-1.31)	1.00 (0.77-1.30)		0.98 (0.75-1.28)	1.00 (0.76-1.32)

All models adjusted for maternal age, maternal height, parity, gestational age and marital status

Model SC (social class) - additionally adjusts only for social class

Model AD (area deprivation) - additionally adjusts only for area deprivation

Model SC+AD - additionally adjusts for both social class and area deprivation

The odds ratios for social class and area deprivation are for the relative index of inequality (RII) calculated for these variables. The odds ratio compares the bottom of the social hierarchy to the top; the larger the RII the larger the inequality.

At all three time points increasing maternal age increased the odds of having an emergency Caesarean section. When additionally adjusting for social class and area deprivation the effect sizes remained unchanged. Increasing maternal height decreased the odds of having an emergency Caesarean section. This was the case for all three time points but the effect was marginally smaller in 1999-2000 than the previous two decades. For parity, at all 3 time points the effect size was similar and compared to having had no previous births the odds of having an emergency Caesarean section decreased as the number of births a mother has had increased. In 1980-81 and 1990-91 there was no difference in the odds of having an emergency Caesarean section for single mothers compared to married mothers, but the odds were increased for those in the "other" category compared to married mothers. In 1999-2000 the odds were increased for both single and others compared to married mothers. For gestational age, at all 3 time points the odds of having an emergency Caesarean section were lower for babies delivered between 37-40 weeks and over 40 weeks compared to those born before 37 weeks; the lowest odds were in the 37-40 weeks group. In 1990-91 and 1990-2000 the effect was stronger than in 1980-81 (Table 4).

Table 3 shows the results for elective Caesarean sections. In 1980-81 women from the most deprived areas were more likely to have an elective Caesarean; this relationship remained significant after adjusting for individual social class (RII = 1.19, 1.03-1.38). This association was considerably stronger among women who had had a previous Caesarean (RII = 1.45, 1.13-1.85). There were no associations between elective sections and either social class or deprivation in 1990-91. In 1999-2000 both social class and area deprivation were associated with elective Caesareans. The odds of having an elective section were lower for women at the bottom of the social class hierarchy than those at the top (RII = 0.87, 0.76-1.00) and also lower in women in the most deprived areas compared to those in the most affluent (RII = 0.85, 0.73-0.99). The significant association with social class was observed in the women with previous sections (RII = 0.81, 0.65-1.00) while the association with deprivation occurred in women with no previous Caesareans (RII = 0.75, 0.58-0.96).

**Table 3:** Relative index of inequality (95% CI) for elective Caesarean sections for social class (SC) and area deprivation (AD), Scotland 1980-2000

	1980-81			1990-91			1999-2000		
	Model SC	Model AD	Model SC+AD	Model SC	Model AD	Model SC+AD	Model SC	Model AD	Model SC+AD
	RII (95%CI)	RII (95%CI)	RII (95%CI)	RII (95%CI)	RII (95%CI)	RII (95%CI)	RII (95%CI)	RII (95%CI)	RII (95%CI)
**All Births**									
Social Class	0.94 (0.83-1.06)		0.91 (0.80-1.03)	1.04 (0.92-1.18)		1.04 (0.92-1.18)	0.85 (0.75-0.97)		0.87 (0.76-1.00)
Area Deprivation		1.17 (1.01-1.34)	1.19 (1.03-1.38)		1.01 (0.88-1.15)	1.00 (0.87-1.14)		0.83 (0.71-0.96)	0.85 (0.73-0.99)
**No previous Caesarean section**									
Social Class	1.06 (0.88-1.27)		1.04 (0.87-1.26)	1.09 (0.86-1.37)		1.08 (0.85-1.37)	0.88 (0.70-1.09)		0.91 (0.73-1.14)
Area Deprivation		1.12 (0.90-1.38)	1.11 (0.89-1.47)		1.04 (0.81-1.34)	1.02 (0.79-1.32)		0.73 (0.57-0.94)	0.75 (0.58-0.96)
**Previous Caesarean section**									
Social Class	0.82 (0.65-1.02)		0.76 (0.61-0.95)	0.92 (0.76-1.11)		0.91 (0.75-1.10)	0.80 (0.65-0.99)		0.81 (0.65-1.00)
Area Deprivation		1.36 (1.07-1.73)	1.45 (1.13-1.85)		1.06 (0.86-1.30)	1.08 (0.88-1.33)		0.92 (0.73-1.16)	0.96 (0.76-1.22)

All models adjusted for maternal age, maternal height, parity, gestational age and marital status

Model SC (social class) - additionally adjusts only for social class

Model AD (area deprivation) - additionally adjusts only for area deprivation

Model SC+AD - additionally adjusts for both social class and area deprivation

The odds ratios for social class and area deprivation are for the relative index of inequality (RII). The odds ratio compares the bottom of the social hierarchy to the top; the larger the RII the larger the inequality.

For both maternal age and height the relationship is similar for elective Caesarean sections to that for emergency Caesarean sections; increasing maternal age increased the odds of having an elective Caesarean section and increasing maternal height decreased the odds of having an elective Caesarean section. For parity, the odds of having an elective Caesarean section were higher for mothers who had had one or two previous births but in 1980-81 were lower for mothers who had had 3 previous births. In 1990-91 and 1999-2000 mothers who had had 3 previous births had increased odds of elective Caesarean section compared to those with no previous births but the odds were lower than for mothers with one or two previous births. At all 3 time points, single mothers were less likely than married mothers to have an elective Caesarean section. There was no difference in the odds of having an elective Caesarean section for those in the "other" category compared to married mothers. Increasing gestational age decreased the odds of having an elective Caesarean section. This was the case for all three time points (Table 4).

**Table 4:** Odds Ratios (95% CI) for maternal factors related to emergency and elective Caesarean sections, Scotland 1980-2000

	EMERGENCY					
	**1980-81**		**1990-91**		**1999-2000**	

	**Maternal factors**	**Model SC+AD**	**Maternal factors**	**Model SC+AD**	**Maternal factors**	**Model SC+AD**

**Maternal age**						
<20	1	1	1	1	1	1
20-24	1.37 (1.25-1.51)	1.39 (1.27-1.53)	1.44 (1.30-1.59)	1.45 (1.31-1.60)	1.61 (1.44-1.79)	1.61 (1.45-1.80)
25-29	1.74 (1.58-1.92)	1.80 (1.63-1.99)	1.89 (1.71-2.09)	1.93 (1.75-2.13)	2.36 (2.12-2.62)	2.38 (2.13-2.65)
30-34	2.31 (2.07-2.57)	2.42 (2.17-2.71)	2.44 (2.19-2.71)	2.52 (2.26-2.81)	3.11 (2.80-3.47)	3.14 (2.81-3.51)
35-39	3.56 (3.08-4.10)	3.74 (3.23-4.32)	3.30 (2.91-3.75)	3.43 (3.02-3.91)	4.19 (3.72-4.72)	4.23 (3.74-4.78)
≥40	5.91 (4.69-7.44)	6.18 (4.90-7.79)	5.00 (4.04-6.19)	5.22 (4.21-6.47)	4.84 (4.01-5.83)	4.88 (4.04-5.91)
**Maternal height**						
<155 cm	1	1	1	1	1	1
155 cm-164 cm	0.50 (0.47-0.53)	0.51 (0.48-0.54)	0.57 (0.54-0.61)	0.58 (0.54-0.61)	0.64 (0.59-0.69)	0.64 (0.60-0.69)
>164 cm	0.30 (0.28-0.33)	0.31 (0.29-0.33)	0.36 (0.34-0.39)	0.37 (0.34-0.40)	0.43 (0.39-0.46)	0.43 (0.40-0.46)
**Parity**						
0	1	1	1	1	1	1
1	0.39 (0.37-0.42)	0.39 (0.36-0.41)	0.35 (0.33-0.37)	0.34 (0.33-0.36)	0.33 (0.31-0.35)	0.33 (0.31-0.35)
2	0.26 (0.23-0.28)	0.25 (0.23-0.28)	0.21 (0.19-0.23)	0.21 (0.19-0.23)	0.20 (0.18-0.22)	0.20 (0.18-0.22)
3+	0.23 (0.20-0.26)	0.22 (0.19-0.25)	0.18 (0.16-0.21)	0.18 (0.16-0.21)	0.21 (0.19-0.24)	0.21 (0.18-0.24)
**Marital status**						
Married	1	1	1	1	1	1
Single	0.94 (0.85-1.04)	0.91 (0.83-1.01)	0.88 (0.82-0.93)	0.85 (0.80-0.91)	1.06 (1.00-1.13)	1.06 (1.00-1.13)
Other	1.29 (1.12-1.48)	1.26 (1.09-1.44)	1.03 (0.94-1.14)	1.01 (0.92-1.11)	1.11 (1.03-1.19)	1.10 (1.03-1.18)
**Gestational age**						
<37 wks	1	1	1	1	1	1
37 wks-40 wks	0.56 (0.51-0.62)	0.56 (0.51-0.62)	0.28 (0.26-0.30)	0.28 (0.26-0.30)	0.30 (0.28-0.33)	0.30 (0.28-0.33)
>40 wks	0.74 (0.66-0.82)	0.74 (0.67-0.83)	0.43 (0.40-0.47)	0.44 (0.40-0.47)	0.48 (0.44-0.52)	0.48 (0.44-0.52)

						
	**ELECTIVE**					

	**1980-81**		**1990-91**		**1999-2000**	

	**Maternal factors**	**Model SC+AD**	**Maternal factors**	**Model SC+AD**	**Maternal factors**	**Model SC+AD**
						

**Maternal age**						
<20	1	1	1	1	1	1
20-24	1.78 (1.48-2.14)	1.78 (1.49-2.14)	1.37 (1.08-1.74)	1.37 (1.08-1.74)	1.88 (1.39-2.54)	1.85 (1.37-2.50)
25-29	2.40 (1.99-2.88)	2.42 (2.01-2.91)	2.07 (1.64-2.62)	2.08 (1.64-2.63)	2.77 (2.07-3.71)	2.68 (2.00-3.60)
30-34	3.51 (2.90-4.24)	3.55 (2.92-4.31)	2.72 (2.14-3.45)	2.74 (2.16-3.48)	3.63 (2.71-4.87)	3.46 (2.58-4.66)
35-39	6.09 (4.93-7.51)	6.17 (4.99-7.62)	3.78 (2.94-4.85)	3.81 (2.96-4.91)	4.96 (3.68-6.68)	4.67 (3.46-6.32)
≥40	10.2 (7.76-13.4)	10.32 (7.86-13.6)	5.52 (4.05-7.52)	5.57 (4.08-7.61)	6.37 (4.56-8.88)	5.99 (4.28-8.37)
**Maternal height**						
<155 cm	1	1	1	1	1	1
155 cm-164 cm	0.45 (0.42-0.48)	0.45 (0.42-0.49)	0.43 (0.40-0.47)	0.43 (0.40-0.47)	0.57 (0.51-0.63)	0.56 (0.51-0.62)
>164 cm	0.31 (0.28-0.34)	0.31 (0.28-0.34)	0.28 (0.25-0.31)	0.28 (0.25-0.31)	0.42 (0.38-0.46)	0.41 (0.37-0.45)
**Parity**						
0	1	1	1	1	1	1
1	1.91 (1.76-2.08)	1.91 (1.75-2.08)	4.25 (3.85-4.68)	4.24 (3.84-4.67)	4.25 (3.84-4.71)	4.30 (3.88-4.76)
2	1.56 (1.41-1.73)	1.55 (1.40-1.72)	4.21 (3.77-4.70)	4.20 (3.76-4.70)	4.26 (3.80-4.79)	4.35 (3.87-4.90)
3+	0.90 (0.78-1.03)	0.89 (0.77-1.02)	2.19 (1.88-2.54)	2.17 (1.87-2.53)	2.83 (2.44-3.27)	2.94 (2.53-3.41)
**Marital status**						
Married	1	1	1	1	1	1
Single	0.78 (0.65-0.94)	0.78 (0.65-0.94)	0.77 (0.69-0.86)	0.77 (0.68-0.86)	0.71 (0.65-0.79)	0.74 (0.67-0.82)
Other	0.99 (0.82-1.19)	0.99 (0.82-1.19)	0.88 (0.78-1.00)	0.88 (0.77-1.00)	0.92 (0.83-1.01)	0.94 (0.85-1.03)
**Gestational age**						
<37 wks	1	1	1	1	1	1
37 wks-40 wks	0.38 (0.34-0.42)	0.38 (0.34-0.42)	0.68 (0.60-0.77)	0.68 (0.60-0.77)	1.10 (0.95-1.27)	1.09 (0.94-1.26)
>40 wks	0.14 (0.12-0.16)	0.14 (0.12-0.16)	0.13 (0.11-0.16)	0.13 (0.11-0.16)	0.17 (0.14-0.21)	0.17 (0.14-0.21)

Maternal factors - adjusts for maternal age, maternal height, parity, gestational age and marital status, only

Model SC+AD - additionally adjusts for both social class and area deprivation

There were differences in the relationships between the maternal confounders, age, parity, marital status and gestational age, in those who had had a previous Caesarean section and those who had had no previous Caesarean section. Multilevel models allow the partitioning of the variance across the 3 levels. There were differences in the variances at Health Board and postcode sector levels between previous and no previous Caesarean section. In 1980-81 and 1999-2000 there was more variation at the Health Board level for previous compared to no previous Caesarean section. At the postcode sector level there was more variation for no previous Caesarean section compared to previous Caesarean section at all time points (Tables 5 and 6).

**Table 5:** Variance estimates (se) from multilevel logistic regression for emergency Caesarean sections for Health Board and Postcode Sector, Scotland 1980-2000

			1980-81		
	Null	Maternal factors	Model SC	Model AD	Model SC+AD
**All Births**					
Health Board	0.053 (0.023)	0.051 (0.022)	0.051 (0.022)	0.055 (0.024)	0.054 (0.023)
Postcode Sector	0.033 (0.007)	0.029 (0.007)	0.029 (0.007)	0.027 (0.007)	0.027 (0.007)

**No previous Caesarean section**					
Health Board	0.023 (0.011)	0.014 (0.007)	0.014 (0.008)	0.013 (0.007)	0.013 (0.007)
Postcode Sector	0.040 (0.009)	0.031 (0.009)	0.030 (0.009)	0.029 (0.008)	0.029 (0.008)

**Previous Caesarean section**					
Health Board	0.439 (0.183)	0.478 (0.198)	0.476 (0.197)	0.484 (0.200)	0.479 (0.199)
Postcode Sector	0.074 (0.041)	0.071 (0.041)	0.069 (0.041)	0.070 (0.041)	0.069 (0.041)

			**1990-91**		
	**Null**	**Maternal factors**	**Model SC**	**Model AD**	**Model SC+AD**

**All Births**					
Health Board	0.037 (0.016)	0.040 (0.017)	0.040 (0.017)	0.038 (0.016)	0.038 (0.016)
Postcode Sector	0.026 (0.006)	0.024 (0.006)	0.023 (0.006)	0.023 (0.006)	0.022 (0.006)

**No previous Caesarean section**					
Health Board	0.034 (0.015)	0.037 (0.016)	0.037 (0.016)	0.035 (0.015)	0.035 (0.015)
Postcode Sector	0.025 (0.007)	0.020 (0.007)	0.020 (0.007)	0.019 (0.007)	0.019 (0.006)

**Previous Caesarean section**					
Health Board	0.040 (0.026)	0.051 (0.027)	0.051 (0.027)	0.051 (0.027)	0.051 (0.027)
Postcode Sector	0.098 (0.035)	0.070 (0.035)	0.071 (0.035)	0.071 (0.035)	0.071 (0.035)

			**1999-2000**		
	**Null**	**Maternal factors**	**Model SC**	**Model AD**	**Model SC+AD**

**All Births**					
Health Board	0.016 (0.007)	0.016 (0.008)	0.016 (0.008)	0.017 (0.008)	0.017 (0.008)
Postcode Sector	0.015 (0.006)	0.012 (0.006)	0.012 (0.006)	0.012 (0.006)	0.012 (0.006)

**No previous Caesarean section**					
Health Board	0.015 (0.007)	0.014 (0.007)	0.014 (0.007)	0.014 (0.007)	0.014 (0.007)
Postcode Sector	0.019 (0.007)	0.017 (0.007)	0.017 (0.007)	0.017 (0.007)	0.017 (0.007)

**Previous Caesarean section**					
Health Board	0.030 (0.019)	0.025 (0.017)	0.024 (0.017)	0.024 (0.017)	0.024 (0.017)
Postcode Sector	0.086 (0.048)	0.095 (0.043)	0.094 (0.043)	0.095 (0.043)	0.094 (0.043)

Null model - contains intercept only

Maternal factors - adjusts for maternal age, maternal height, parity, gestational age and marital status

Model SC (social class) - additionally adjusts only for social class

Model AD (area deprivation) - additionally adjusts only for area deprivation

Model SC+AD - additionally adjusts for both social class and area deprivation

**Table 6:** Variance estimates (se) from multilevel logistic regression for elective Caesarean sections for Health Board and Postcode Sector, Scotland 1980-2000

			1980-81		
	Null	Maternal factors	Model SC	Model AD	Model SC+AD
**All Births**					
Health Board	0.728 (0.281)	0.707 (0.274)	0.707 (0.274)	0.690 (0.267)	0.688 (0.266)
Postcode Sector	0.026 (0.010)	0.03 (0.011)	0.03 (0.011)	0.028 (0.011)	0.028 (0.011)

**No previous Caesarean section**					
Health Board	0.809 (0.325)	0.809 (0.326)	0.808 (0.325)	0.794 (0.320)	0.795 (0.320)
Postcode Sector	0.064 (0.023)	0.079 (0.025)	0.079 (0.025)	0.078 (0.025)	0.078 (0.025)

**Previous Caesarean section**					
Health Board	0.988 (0.386)	1.099 (0.428)	1.102 (0.429)	1.044 (0.408)	1.037 (0.405)
Postcode Sector	0.053 (0.028)	0.032 (0.028)	0.034 (0.028)	0.028 (0.028)	0.029 (0.028)

			**1990-91**		
	**Null**	**Maternal factors**	**Model SC**	**Model AD**	**Model SC+AD**

**All Births**					
Health Board	0.038 (0.018)	0.023 (0.011)	0.023 (0.011)	0.023 (0.011)	0.023 (0.011)
Postcode Sector	0.02 (0.010)	0.011 (0.010)	0.011 (0.010)	0.011 (0.010)	0.011 (0.010)

**No previous Caesarean section**					
Health Board	0.054 (0.029)	0.031 (0.019)	0.03 (0.019)	0.03 (0.019)	0.03 (0.019)
Postcode Sector	0.081 (0.038)	0.063 (0.019)	0.063 (0.037)	0.063 (0.037)	0.063 (0.037)

**Previous Caesarean section**					
Health Board	0.058 (0.028)	0.040 (0.021)	0.040 (0.021)	0.039 (0.020)	0.039 (0.020)
Postcode Sector	0.040 (0.021)	0.035 (0.023)	0.035 (0.023)	0.034 (0.023)	0.034 (0.023)

			**1999-2000**		
	**Null**	**Maternal factors**	**Model SC**	**Model AD**	**Model SC+AD**

**All Births**					
Health Board	0.037 (0.018)	0.03 (0.015)	0.031 (0.015)	0.034 (0.016)	0.034 (0.016)
Postcode Sector	0.087 (0.015)	0.066 (0.014)	0.065 (0.014)	0.063 (0.014)	0.062 (0.014)

**No previous Caesarean section**					
Health Board	0.123 (0.058)	0.100 (0.048)	0.100 (0.048)	0.113 (0.053)	0.112 (0.053)
Postcode Sector	0.211 (0.043)	0.153 (0.038)	0.151 (0.038)	0.144 (0.038)	0.144 (0.037)

**Previous Caesarean section**					
Health Board	0.072 (0.035)	0.063 (0.031)	0.065 (0.032)	0.066 (0.032)	0.066 (0.032)
Postcode Sector	0.106 (0.030)	0.095 (0.032)	0.095 (0.032)	0.094 (0.032)	0.094 (0.032)

Null model - contains intercept only

Maternal factors - adjusts for maternal age, maternal height, parity, gestational age and marital status

Model SC (social class) - additionally adjusts only for social class

Model AD (area deprivation) - additionally adjusts only for area deprivation

Model SC+AD - additionally adjusts for both social class and area deprivation

For both emergency and elective Caesareans, at all time points there was variation between Health Boards and postcode sectors that could not be explained by individual social class, area deprivation or the other risk factors (Tables 5 and 6). The total area variance unexplained following adjustment for individual social class and area deprivation in 1980-81, 0.081 (= 0.054 + 0.027), suggests that about 2.4% of the unexplained variation may be associated with area differences, [27] with most of this arising due to differences between Health Boards.

For both emergency and elective Caesarean sections the variance at the Health Board level reduced substantially from 1980-81 to 1999-2000; for example, for emergency sections the variance reduced from 0.054 to 0.017. For emergency Caesarean sections, at all three time points, adjusting for maternal factors and additionally social class and area deprivation makes little difference to the variation between Health Boards (Table 5). For elective sections there was a substantial reduction in the variance at the Health Board level from 1980-81 to 1990-91. Although the variation between Health Boards was very small by 1999-2000, adjusting for maternal factors reduced the variance between Health Boards from 0.037 to 0.030. Further adjustment for social class and area deprivation did not reduce this variance (Table 6).

## Discussion

Both individual social class and area deprivation are independently associated with Caesarean sections in Scotland. Inequalities have disappeared for emergency sections whilst appearing for elective sections. In 1980-81 and 1990-91 women with lower socioeconomic position were more likely to have an emergency Caesarean than women with higher socioeconomic position, but there were no gradients by social class or area deprivation by 1999-2000.

It is clear that the patterns of inequalities differ for emergency and elective sections. The overall rate of Caesarean sections was not associated with area deprivation in Scotland in the mid 1990s [15]. However, by considering the two separately we found area deprivation to be associated significantly (at different times) with both emergency and elective Caesareans, indicating the importance of considering the two separately.

The main strength of this study is the use of two different indicators of socioeconomic status: individual social class and area deprivation. Area deprivation has the advantage that it is more widely available than individual social class in routine data. Studies in England and Wales investigating inequalities in birthweight have shown that area deprivation performs as well as or better than individual social class in describing the extent of inequalities in the population [28,29]. However, when assessing inequalities in stillbirths individual social class was shown to be a better predictor than area deprivation [30]. In this study we found that for emergency and elective Caesareans including both socioeconomic measures in the models only slightly attenuated the effect of each suggesting that they may capture different dimensions of deprivation; individuals with low occupational social class may live in affluent areas, for example.

In 1980-81 emergency Caesarean section rates were highest among the disadvantaged groups, whether defined by area deprivation or individual social class. Over time these differences have disappeared; by 1999-2000 there was no evidence of a social gradient in emergency Caesarean section rates. This is perhaps counter-intuitive given the consistently poorer obstetric performance of women from lower social classes [24]. Rising emergency Caesarean rates have been shown to be related to increasing maternal age [7]. Mothers in the highest social class have been shown to be older than those in the lower social classes and this relationship was observed over the 3 time periods; [31] however, all our analyses adjusted for maternal age.

In 1999-2000 there was a significant association between area deprivation and elective Caesareans. Women living in the most deprived areas were less likely to have elective Caesareans than women in the most affluent areas; this concurs with finding in England in 2001-2002 [13] and 1996-2000 [14]. Neither of these studies had time series data and could not examine trends. Our study shows this association represents a change from the early 1980s, when elective sections (as with emergency sections) were more common in deprived areas. There was also a significant association between elective Caesareans and individual social class, with women at the bottom of the social class hierarchy less likely to have an elective Caesarean than women at the top. These two dimensions of disadvantage were independent of each other, were approximately equal in size and showed little sign of attenuation upon adjustment for the other.

The individual social class variable, and resulting relative index of inequality, is derived from the occupation of the father or that of the mother if the father is not present. Both maternal and paternal social class have been shown to be related to adverse pregnancy outcomes; [32] however, there are issues with assigning maternal social class [33]. The Register General's social class was constructed in relation to men's occupations and therefore it may not be appropriate to use mother's occupation. Additionally women are more likely to be looking after the home and family or never to have worked and so have no occupation and therefore can not be assigned their own social class.

The magnitude of the deprivation effect on elective Caesarean sections in Scotland in 1999-2000 (RII = 0.83; 95% CI = 0.71-0.96) is comparable to that seen in England in 2001-2002 (OR comparing most deprived to least deprived quintile = 0.86; 95% CI = 0.82-0.89) [13]. However, that analysis omitted the independent effect of individual social class. Since women of lower social status are more likely to live in more deprived areas - the social class of the head of household is one of the four factors that comprise the Carstairs index - the use of one socioeconomic indicator alone may underestimate the effect of the inequality. The RIIs presented in tables 2 and 3 are odds ratios and as such are multiplicative; table 3 therefore suggests that the odds of a woman of the highest social status from the least deprived area receiving an elective Caesarean in 1999-2000 are 35% higher than those for a woman of the lowest social class living in the most deprived area, *ceteris paribus*.

By using the RII we were able to compare social class inequalities at each time point and also compare inequalities by both individual social class and area deprivation directly. The area deprivation quintile, based on the postcode of residence of the mother at the time of birth, assumes that all the residents of the geographical areas are socially homogeneous [19]. By testing for the interaction between social class RII and the area deprivation quintiles we were able to show that inequalities by social class were constant across all quintiles of deprivation.

An example of the differential effects of individual social class and area deprivation is given by the analysis of elective sections stratified by previous Caesarean section. Such stratification adds insight to the analysis given the importance of previous Caesarean section as a predictor of subsequent sections [34]. In 1999-2000 women living in deprived areas were significantly less likely to receive a first section whilst this was not significantly affected by social class. On the other hand, repeat sections were more common among women from higher social classes and were unaffected by area deprivation. Clearly the two measures of socioeconomic circumstances are manifesting themselves differently. A deeper understanding of socioeconomic gradients in the provision of Caesarean sections and the mechanisms that create them - for example, the contribution of maternal request or negotiation [35,36] - requires a detailed analysis of potential differences between the two measures.

The provider will exert an influence on Caesarean section rates due to differences in culture, policy, experience and facilities. The use of multilevel models allows for the partitioning of the variance across the different levels. For both emergency and elective Caesarean sections there was substantial unexplained variation between Health Boards, reflecting differences between hospitals as well as differences between populations. Significant variation among Health Boards in the rates of emergency and elective Caesareans throughout the 1990s in Scotland has been reported elsewhere and standardisation for maternal age and deprivation had little impact on these rates [37]. We also found that adjustment for other known risk factors for Caesarean sections including individual social class did not reduce the variation between Health Boards in any of the time periods.

## Conclusions

It is clear that maternal social class and area deprivation are different indicators of socioeconomic status which exhibit independent effects on the probability of a woman receiving a Caesarean section. Between 1980 and 2000 in Scotland the disappearance of the social gradient for emergency Caesarean sections (with higher rates among more disadvantaged women) may reflect a decrease in the socioeconomic gradient in obstetric complications indicating the need for emergency intervention. It is less clear why a socioeconomic gradient should emerge for elective Caesarean sections (with lower rates among more disadvantaged women); the factors leading to the clinical decision to plan a Caesarean delivery are not routinely recorded but need to be understood to assess whether the provision of healthcare is equitable.

## Competing interests

The authors declare that they have no competing interests.

## Authors' contributions

LF contributed to the study concept and analysed and interpreted the data and drafted the manuscript; RD participated in the analysis and interpretation of the data; AHL contributed to the study concept and the interpretation of the data. All authors contributed to the critical revision of the article for important intellectual content and have read and approved the article.

## Pre-publication history

The pre-publication history for this paper can be accessed here:

http://www.biomedcentral.com/1471-2458/11/330/prepub
